# The Relationship Between Childhood Trauma and Perfectionism in Obsessive Compulsive Disorder

**DOI:** 10.1192/j.eurpsy.2026.11597

**Published:** 2026-07-31

**Authors:** F. M. Topur, E. Porgalı Zayman

**Affiliations:** 1 Psychiatry, Siirt Eğitim ve Araştırma Hastanesi, Siirt; 2 Psychiatry, İnönü University, Malatya, Türkiye

## Abstract

**Introduction:**

Cognitive models of obsessive-compulsive disorder (OCD) have indicated that perfectionism constitutes one of the dysfunctional cognitive patterns contributing to the disorder’s etiology. Perfectionism represents a multifaceted psychological construct that is frequently associated with childhood trauma.

**Objectives:**

The present study aimed to investigate the specific impact of childhood traumatic experiences and perfectionism tendencies on the severity of OCD symptoms.

**Methods:**

This case–control study included 76 outpatients who presented to the Department of Mental Health and Diseases at Turgut Özal Medical Center and were diagnosed with OCD according to the Diagnostic and Statistical Manual of Mental Disorders, Fifth Edition criteria. The control group consisted of 80 individuals with no prior history of psychiatric disorders, who were comparable to the patient group in terms of sociodemographic characteristics. Both groups completed a series of assessment instruments, including the Yale–Brown Obsessive–Compulsive Scale – Self-Report Form, the Frost Multidimensional Perfectionism Scale, and the Childhood Trauma Questionnaire.

**Results:**

When compared with the control group, the patient group demonstrated significantly higher scores in the perfectionism subdimensions of concern over mistakes, parental criticism, and doubt about actions, as well as in maladaptive perfectionism and total perfectionism scores. In addition, the patient group showed significantly higher scores in emotional abuse, emotional neglect, physical abuse, and total childhood trauma compared to the control group.A significant positive correlation was observed between the total childhood trauma score and the perfectionism subdimensions of concern over mistakes, parental criticism, and doubt about actions. Furthermore, OCD symptoms also demonstrated significant positive correlations with the same perfectionism subdimensions.In the organization subdimension, a non-significant negative correlation was observed, which appeared only within the patient group.

**Conclusions:**

The observation that significant positive relationships among childhood trauma, OCD symptom severity, and specific perfectionism subdimensions were also present in the control group suggests that these associations may be more closely related to symptom severity rather than being disorder-specific. On the other hand, the non-significant negative correlation observed in the organization subdimension, which was present only in the patient group, appears to reflect an OCD-specific characteristic. This finding implies that the absence of adaptive perfectionism may play a role in the onset or maintenance of psychopathology in individuals with OCD. Overall, the study emphasizes the need for future research with larger samples and causal model testing to better elucidate the nature of these complex relationships.

**Disclosure of Interest:**

None Declared

